# Supplementary material for: Association of the incidence of atopic dermatitis until 3 years old with climate conditions in the first 6 months of life: Japan Environment and Children’s Study (JECS)
Source: PLoS One. 2022 May 6;17(5):e0268204. doi: 10.1371/journal.pone.0268204 (PMC9075629; doi:10.1371/journal.pone.0268204)
Supplement: S1 Table — (DOCX) [file pone.0268204.s001.docx]

**S1 Table. Adjusted HRs (95% confidence intervals) of atopic dermatitis for a low vs. high mean climate condition from birth to 6 months.**

| Low vs. high value | Cut-off value for high or low | Adjusted HR |
| --- | --- | --- |
| Temperature, °C | 15.8 | 1.19 (1.14, 1.24) |
| Maximum temperature, °C | 20.2 | 1.18 (1.13, 1.23) |
| Minimum temperature, °C | 12.1 | 1.18 (1.14, 1.23) |
| Precipitation amount, mm | 148 | 1.14 (1.08, 1.21) |
| Sunshine duration, hours | 166 | 1.05 (0.998, 1.10) |
| Sunshine percentage, % | 45 | 0.91 (0.86, 0.96) |
| Solar radiation quantity, MJ/m2 | 13.8 | 1.15 (1.10, 1.20) |
| Vapor pressure, hPa | 14.4 | 1.17 (1.12, 1.22) |
| Atmospheric pressure, hPa | 1,006 | 0.81 (0.76, 0.87) |
| Humidity, % | 69 | 1.13 (1.08, 1.19) |
| Wind velocity, m/s | 3.1 | 0.98 (0.90, 1.07) |
| Low temperature and low vapor pressure |  | 1.19 (1.14, 1.24) |
| Low temperature and high vapor pressure |  | 0.99 (0.79, 1.26) |
| High temperature and low vapor pressure |  | 0.99 (0.89, 1.10) |
| High temperature and high vapor pressure |  | Reference |
| Low maximum temperature and low vapor pressure |  | 1.19 (1.14, 1.24) |
| Low maximum temperature and high vapor pressure |  | 1.04 (0.86, 1.25) |
| High maximum temperature and low vapor pressure |  | 1.03 (0.93, 1.14) |
| High maximum temperature and high vapor pressure |  | Reference |
| Low minimum temperature and low vapor pressure |  | 1.19 (1.14, 1.24) |
| Low minimum temperature and high vapor pressure |  | 1.11 (0.84, 1.46) |
| High minimum temperature and low vapor pressure |  | 1.02 (0.91, 1.13) |
| High minimum temperature and high vapor pressure |  | Reference |

The hazard ratio was adjusted for a maternal and paternal history of allergy and the prefecture of birth. HR, hazard ratio.
