# Supplementary figures and images for: Association of the incidence of atopic dermatitis until 3 years old with climate conditions in the first 6 months of life: Japan Environment and Children’s Study (JECS)
Source: PLoS One. 2022 May 6;17(5):e0268204. doi: 10.1371/journal.pone.0268204 (PMC9075629; doi:10.1371/journal.pone.0268204)

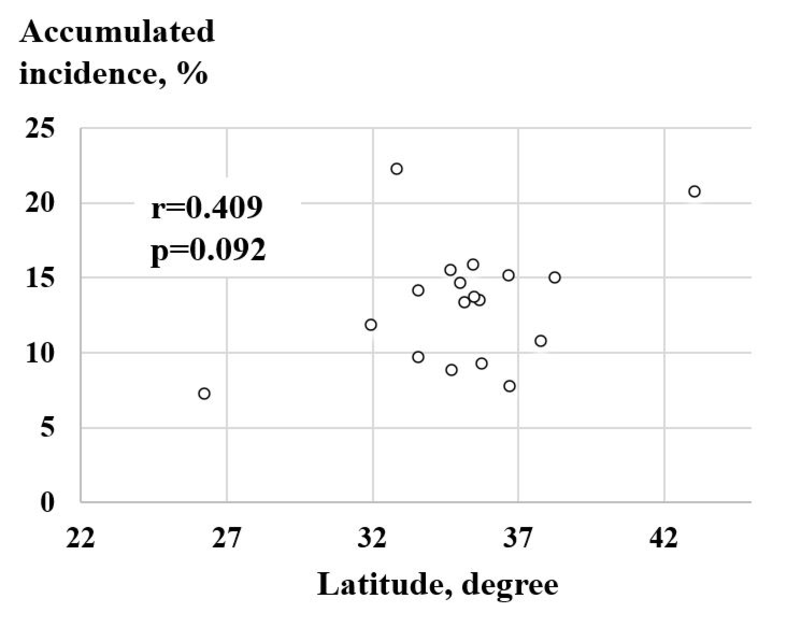

Supplement: S1 Fig — Owing to the small number of participants, data from Shiga Prefecture were included with those of the neighboring Kyoto Prefecture. (TIF) [file pone.0268204.s002.tif]
